# Cellular-Membrane-Derived Vesicles for Cancer Immunotherapy

**DOI:** 10.3390/pharmaceutics16010022

**Published:** 2023-12-22

**Authors:** Xiaoyu An, Yun Zeng, Chao Liu, Gang Liu

**Affiliations:** 1 State Key Laboratory of Molecular Vaccinology and Molecular Diagnostics, National Institute of Diagnostics and Vaccine Development in Infectious Diseases, Center for Molecular Imaging and Translational Medicine, School of Public Health, Xiamen University, Xiamen 361102, China; 21620200156469@stu.xmu.edu.cn; 2 State Key Laboratory of Stress Biology, Fujian Provincial Key Laboratory of Innovative Drug Target Research, School of Pharmaceutical Sciences, Xiamen University, Xiamen 361102, China; 3 School of Life Sciences, Xiamen University, Xiamen 361102, China; 4 Department of Pharmacy, Xiamen Medical College, Xiamen 361023, China; zengyun163@163.com; 5 Shenzhen Research Institute of Xiamen University, Shenzhen 518000, China

**Keywords:** cellular-membrane-derived vesicles, cancer immunotherapy, nanomaterials, drug delivery, combination therapy

## Abstract

The medical community is constantly searching for new and innovative ways to treat cancer, and cellular-membrane-derived artificial vesicles are emerging as a promising avenue for cancer immunotherapy. These vesicles, which are derived from mammal and bacteria cell membranes, offer a range of benefits, including compatibility with living organisms, minimal immune response, and prolonged circulation. By modifying their surface, manipulating their genes, combining them with other substances, stimulating them externally, and even enclosing drugs within them, cellular vesicles have the potential to be a powerful tool in fighting cancer. The ability to merge drugs with diverse compositions and functionalities in a localized area is particularly exciting, as it offers a way to combine different immunotherapy treatments for maximum impact. This review contains information on the various sources of these vesicles and discusses some recent developments in cancer immunotherapy using this promising technology. While there are still obstacles to overcome, the possibilities for cellular vesicles in cancer treatment are truly exciting.

## 1. Introduction

Cancer, marked by uncontrollable cell growth, arises from genetic mutations. Evading the immune system, cancer cells flourish, forming tumors or spreading. They circumvent detection by producing immune-suppressing substances, altering surface antigens, and creating an environment hostile to immune responses. Cancer has been a major challenge for the medical community over the past few decades [1,2]. The immune system, a powerful defense network, is equipped to identify and eliminate these aberrant cells. However, cancer cells employ various strategies to evade detection or suppression by the immune system, thus forming barriers that facilitate their survival and progression [3,4]. Recently, cancer immunotherapy has attracted tremendous attention owing to its advantages: (1) Targeted approach: immunotherapy can specifically identify and attack cancer cells, potentially minimizing damage to healthy cells. (2) Long-term response: it has the potential to create long-lasting effects, with the immune system remembering and continuing to target cancer cells even after treatment ends. (3) Reduced side effects: compared to traditional treatments like chemotherapy, immunotherapy often causes fewer severe side effects [5,6,7]. Chimeric antigen receptor (CAR) T-cell therapy redirects T cells to target and destroy specific antigen-expressing cells, bypassing the MHC receptor for potent anti-tumor action [8]. Immune checkpoint blockade (ICB) therapies function by inhibiting immune checkpoints, like those on T cells and cancer cells, which normally regulate immune responses, ultimately allowing the immune system to attack the tumor [9]. Tumor vasculatures (TAs) are a hallmark of cancer. Improved outcomes in cancer immunotherapy, particularly with ICB, are observed when vessel normalization is achieved [10]. Although these approaches show great promise in the field and have demonstrated significant efficacy and safety advantages compared to traditional treatments, they also face challenges such as low response rates, vulnerability to relapse, and cytokine release syndrome (CRS). 

Biomaterials are substances engineered to interact with biological systems for therapeutic or diagnostic purposes. Nanotechnology, on the other hand, deals with the manipulation and application of materials and structures at the nanoscale [11,12]. Fortunately, the development of biomaterials and nanotechnology offers promising solutions for cancer immunotherapy [13,14,15]. Due to the development of nanomaterials, nanovehicles offer several advantages in various applications, such as controlled drug release, diverse chemical modifications, and high yield. For example, nanoparticles possessing magnetic properties and nanogels responsive to temperature enable targeted drug release, size alteration, transitioning between sol and gel states, as well as magnetic hyperthermia, specifically at tumor locations [16,17,18,19]. Cellular vesicles, which share membrane functions and characteristics with parental cells [20], show superior biocompatibility, lower immunogenicity and toxicity, an extended circulation period, and innate targeting capability compared to traditional nanodrugs [21,22,23].

Numerous studies have documented the use of vesicles derived from red blood cells [24], dendritic cells [25] natural killer cells [26] cancer cells [27], etc., in cancer treatment. This review aims to provide an extensive summary of the diverse applications of cell-derived vesicles and their recent advancements in cancer treatment. We will explore different cellular vesicles and their unique features, originating from distinct sources. Subsequently, we will examine thoroughly the current approaches for cellular vesicles in fighting tumors. In conclusion, we shall explore the utilization of cellular vesicles for drug administration and address the challenges associated with using cellular vesicles in cancer treatment.

## 2. Cellular Vesicles of Diverse Sources Cells

**Figure 1:**
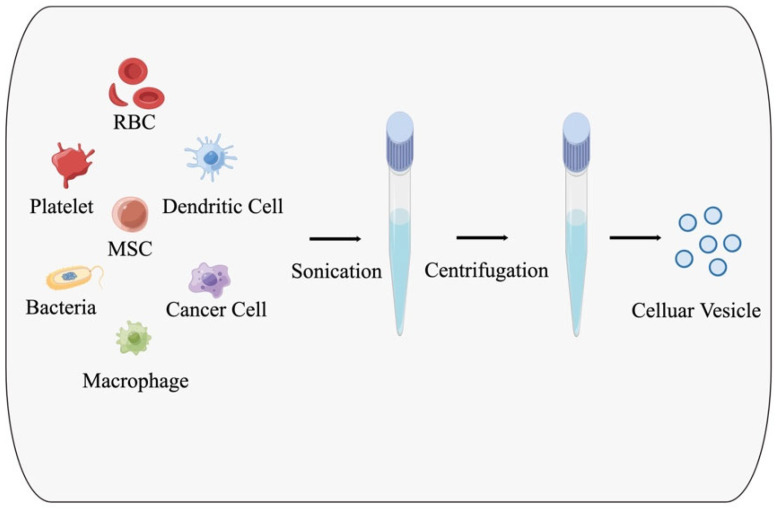
A diagram of vesicles derived from cells.

Nanosized cellular vesicles are typically generated through external forces acting on cell membranes, such as sonication, hypotonic solution exposure, and/or repeated freeze–thaw cycles, which cause the cytoplasm to swell and become susceptible to fragmentation. Subsequently, the vesicles are collected through a multistep density process. These vesicles acquire specific functions based on the unique attributes of their parent cells. Our primary focus is to delve into a comprehensive exploration of commonly encountered cell types and the unique traits manifested by the membrane vesicles they produce [28]. This offers unique opportunities for drug delivery in cancer immunotherapy using different types of cells as carriers. Figure 1 illustrates the various types of cells that can be used in this approach.

### 2.1. Erythrocyte

Erythrocytes are also known as red blood cells (RBCs). Over 99% of blood cells are erythrocytes, which transport oxygen through the circulatory system to tissues [24]. Extensive research has been conducted on their possible uses as microspheres for drug delivery due to their compatibility with living organisms and their ability to break down naturally [29]. In addition, erythrocytes have a significantly extended duration in the bloodstream, and the RES (reticuloendothelial system) can quickly eliminate aged and incompatible red blood cells without generating harmful byproducts. 

Wang and his team have taken advantage of these benefits to develop different formulations for transporting substances to prevent and cure specific forms of tumors. They loaded tumor antigens and anti-PD-L1 into RBC membranes as a cancer vaccine, which effectively activated T cells and prompted an immune response in the spleen through antigen-presenting cells (APCs) [30,31]. In 4T1 and B16F10 tumor models, combining the nanovaccine with anti-PD-L1 suppressed tumor growth, providing new perspectives on cancer treatment and the uses of RBC membranes.

However, red blood cells face numerous challenges when it comes to using them for drug delivery. The RES can rapidly eliminate modified or encapsulated red blood cells due to their morphological and functional alterations. Additionally, RBCs cannot pass through the vascular endothelium to release medicinal substances at damaged tissue sites, making it challenging to target non-RES agents with RBC carriers in areas experiencing cancer [32,33].

### 2.2. Platelet

Platelets are an important component of the bloodstream, as they play a crucial role in preventing bleeding. They have the ability to activate and adhere to wounded regions, which makes them essential in the emergence and progression of various ailments, including cancer and atherosclerosis [34,35]. In 2017, Wang and colleagues proposed the use of platelets as transporters to modify the presence of anti-PD-L1 on their exterior. This was carried out to hinder the recurrence and metastasis after surgical extraction. To test this idea, they conducted experiments on mice with 4T1 and B16F10 tumor models. The results showed that releasing the anti-PD-L1 antibody through the platelet system greatly increased the survival rate of mice and decreased the rate at which cancer regrows. Based on this study, immune checkpoint antibodies could be transported to remaining tumor cells through the platelet system they created, successfully inhibiting tumor reappearance following ablation [36].

### 2.3. Immune Cell

The immune system relies on immune cells to perform a vital function by assisting in the body’s protection against infections and diseases. There are various types of cells, including lymphocytes, macrophages, dendritic cells (DCs), neutrophils, and natural killer cells (NK cells) [37,38,39]. These cells can be used in various drug delivery systems, providing unique benefits that help overcome certain challenges associated with alternative treatments.

Macrophages and dendritic cells are proficient antigen-presenting cells (APCs) that express MHC-II, co-stimulatory molecules, and pattern recognition receptors. Macrophages can be found in every organ and play a critical role in the body’s natural defense system [40]. They can attract drug carriers through inflammation-based chemotaxis, allowing them to facilitate the buildup of these carriers in tumor tissues that are persistently inflamed [41,42]. Researchers observed that nanoparticles encapsulated within macrophage J774 cell membranes displayed a pattern of delayed liver accumulation and maintained their structural integrity for 40 min, which was twice as long as naked nanoparticles. Additionally, when adherent particles were coated with macrophage membranes, a remarkable 25% of these particles managed to evade phagocytosis by Kupffer cells, a significantly higher percentage compared to the roughly 9% evasion rate seen in uncoated nanoparticles. Furthermore, employing macrophage vesicles for drug delivery resulted in a two-fold increase in particle density at tumor sites [43]. Dendritic cells also play a crucial role in stimulating both humoral and cellular immune responses by presenting antigens to T cells [44,45]. They express MHC-I and MHC-II molecules, which enable them to engage with proteins and stimulate T cells, thereby triggering an anticancer immune response [46]. These immune cells offer promising opportunities for drug delivery and cancer therapy.

Neutrophils in cancer provide both support and impediment to tumor growth in the tumor setting [47]. This cell type is highly effective in targeting brain tumors, specifically due to its extraordinary ability to cross the blood–brain barrier [48]. When a tumor is removed, local inflammation occurs, and inflammatory cytokines like tumor necrosis factor (TNF)-α are released. This activation causes neutrophils to move towards the inflamed brain’s surroundings [49]. Xue and colleagues have taken advantage of this process and used neutrophils to enclose liposomes loaded with pentatonix (PTX) [50]. These liposomes can be administered intravenously and relocate to the inflamed surroundings. When they arrive at the tumor site, the liposomes release PTX, which has cytotoxic effects and can prevent tumor recurrence following surgery.

NK cells are a category of lymphocytes that are part of the innate immune system [51]. They demonstrate the capacity to eradicate various types of cancer cells and are particularly proficient in eliminating cells that have metastasized to other parts of the body. However, the effectiveness of these treatments can be limited when dealing with sizable compact neoplasms [52,53]. Artificially engineered NK cells, developed by Zhang and colleagues, can enhance the efficiency of solid tumor treatment by combining them with photothermal therapy. These cells have the ability to identify and eliminate abnormal cells without being restricted by MHC or pre-sensitization, making them the most effective subset of immune cells for surveilling and eradicating cancer cells in the body [54,55]. By combining metal ions and organic dyes, they produced innovative 2D coordination nanosheets (CONASHs) through self-assembly. The CONASHs demonstrated remarkable photothermal conversion both in vivo and in vitro after treatment with PEI and DNAzyme [56].

### 2.4. Stem Cell

Mesenchymal stem cells (MSCs) are a diverse group of cells that possess exceptional qualities of self-renewal and ongoing differentiation [57]. To date, several studies have revealed that MSCs are recruited to tumors and play an important role in the regulation of both solid and hematological malignancies [58]. Researchers have taken advantage of the innate capability of MSCs to actively move towards cancerous locations. By incorporating paclitaxel (PTX) and lactide-co-glycolide (PLGA), the MSCs increased apoptosis and necrosis areas within tumor tissues to suppress the primary tumor growth and attenuate the toxic side effects [59].

### 2.5. Cancer Cell

The presence of particular antigens in vesicles originating from cancer cells enhances their ability to target tumors of the same type. By processing these vesicles, nanovaccines can be created to enhance the maturation of DCs and facilitate antigen extraction. Activating T cells and initiating various immune responses occur when APCs present the antigen on the surface of cancer cells after capturing the antigen. The presented strategy provides innovative concepts and techniques for cancer immunotherapy [60]. Moreover, Zhang and colleagues reported a biomimetic nanoparticle that contained a photosensitizer (ICG) and a hydrophobic medication (HCPT), both of which were enclosed in 4T1 tumor cell membranes. The enriched ICG not only enabled photothermal therapy but also enhanced the effectiveness of the hydrophobic medication. It was found that biomimetic nanoparticles inhibited tumor growth effectively in breast cancer models while also maintaining excellent biological safety [61].

### 2.6. Bacteria

The presence of a cell wall distinguishes Gram-positive bacteria from Gram-negative bacteria and serves as their main differentiating factor. Bacteria have long been used to treat cancer, with one of their key benefits being their exceptional phagocytosis and their role as pathogen-associated molecular patterns (PAMPs). In response to interactions with pattern recognition receptors (PRRs), they initiate a sequence of immune reactions. In addition, bacteria have the ability to transport therapeutic agents and specifically target APCs. Several studies have shown that bacteria have the ability to specifically inhibit oxygen-deprived areas within tumors, effectively impeding the growth of a tumor [62,63]. According to Leventhal and colleagues, the *E. coli* strain SYNB1891 was created by employing synthetic biology techniques. The modified strain possessed the capacity to specifically target APCs located within tumors and activate the stimulator of the interferon genes (STING) pathway, consequently inducing additional innate immune responses. Mouse tumor models were treated with SYNB1891, and an immune response was observed, as well as the induction of immune memory [64]. However, there are challenges linked to the utilization of bacterial vesicles in cancer immunotherapy: bacterial vesicles have the potential to trigger immune responses or induce adverse reactions in certain individuals, thus posing safety risks, and pre-existing or induced bacterial-specific immune memory may interfere with therapeutic efficacy. Studies have reported that *E. coli* OMVs provoke a sepsis-like inflammatory response, activate endothelial cells and platelets, leading to a procoagulant state, and further induce cardiac dysfunction [65].

## 3. Applications and Approaches Using Cellular Vesicles

### 3.1. Manipulation of Cellular Vesicles through Genetic Engineering

**Figure 2:**
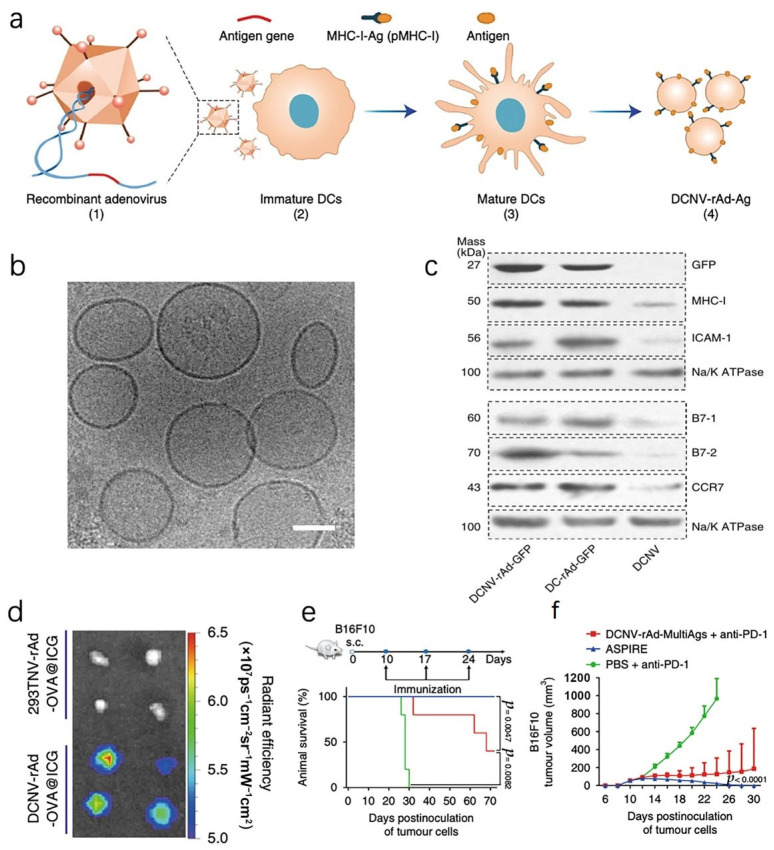
Genetic engineering of DCs. (**a**) A diagram showing the procedure for creating ASPIRE. (**b**) Cryo-electron microscopy imaging of cellular vesicles; the scale bar is 50 nm. (**c**) Western blot analysis of membrane proteins from DCNV-rAd-GFP cells shows similar amounts of proteins as those on the parental cells. (**d**) Fluorescent signals detected in the draining of inguinal LNs. (**e**) Curves of survival for mice treated and untreated. (**f**) The tumor progression graphs of B16F10. Reproduced with permission [69]. Copyright 2022, Nature Nanotechnology.

Through gene editing, cells can express proteins specifically promoting APC presentation [24,66], regulating the TME [67], activating T cells, and inducing the release of tumor-associated antigens (TAAs) that cause immunogenic cell death (ICD) [68]. The ability of their extruded vesicles to maintain this functionality to promote DCs’ presentation and activate T cells has been demonstrated by Liu et al. in their explanation of ASPIRE, a cancer immunotherapy technique. This technique combines the self-presentation of antigens with immunoreversal by utilizing genetically modified nanovesicles on the cell surface. The ASPIRE cellular vesicles are created from DCs which are infected with recombinant adenovirus. The program process (Figure 2a) simultaneously anchors peptide–major histocompatibility complex class I (pMHC-I), B7 co-stimulatory molecules, and an anti-PD1 antibody. As shown in Figure 2d, this method can significantly improve antigen delivery to lymphoid organs. Endogenous and depleted T cells are activated, and established tumors can be effectively eliminated (Figure 2e) [69]. Another example is the transfection of the signal regulatory protein alpha (SIRPα) and PD-1 sequences into tumor cells. These modified cells produced vesicles that, when fused, exhibited elevated levels of both proteins on their surface, effectively blocking both innate and adaptive immune checkpoints at the same time [70].

**Figure 3:**
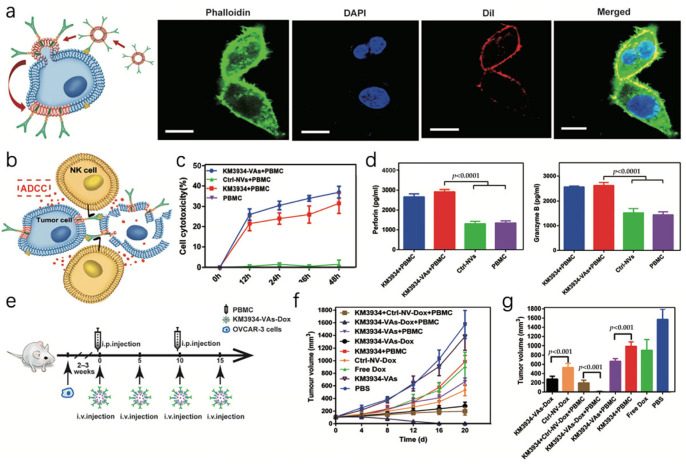
Cellular vesicles exhibit complete monoclonal antibodies. (**a**)After incubating HepG2 cells with DiI-labeled hGC33 cellular vesicles, a display of energy-dependent membrane fusion mediated by antibodies was demonstrated; the scale bar is 20 μm. (**b**) The cytotoxic activity mediated by hGC33-Bio-MVs involves antibody-dependent cellular cytotoxicity (ADCC). (**c**) The KM3934-VAs mediate the cytotoxicity activity of ADCC. (**d**) In a 24 h time period, ELISA was used to assess NK cell activation and cytokine production (perforin and granzyme B). (**e**) Illustration depicting the integration of various therapeutic approaches. (**f**) Tumor expansion was assessed throughout the duration, and the volume of tumors in each group was computed on the 20th day. (**g**) Tumor volume of each group was calculated on the 20th day. Reproduced with permission [71]. Copyright 2019, Wiley-VCH.

Furthermore, cellular vesicles were additionally modified to exhibit antibodies for cancer cell tracking and antibody-dependent cellular cytotoxicity for elimination [71]. A monoclonal antibody (anti-GPC3 or hGC33) was genetically engineered onto HEK293T cell membranes to target HCC. The cells efficiently took up the enclosed components via the antibody-facilitated fusion of cell membranes that require energy and the transfer of antibodies to a specific cell membrane (Figure 3a). NK cells were recruited and aggregated within the microenvironment due to the Fc domain present in tumor cells, facilitating antibody-dependent cellular cytotoxicity (ADCC) and tumor necrosis mediation. As a result of the observation above, genetically modified antibodies can maintain structure and morphology on cell membranes (Figure 3b). In the experiment, healthy human PBMCs were injected into animals intraperitoneally as effector cells (Figure 3e). The tumor sizes of every group were observed for the following 20 days, and KM3934-VAs-Dox displayed the most significant tumor inhibition among the chemotherapy groups (Figure 3f,g). Additionally, cellular vesicles were utilized to treat hepatic cancer by extruding CAR-T cells [72].

### 3.2. Hybridization of Cellular Vesicles

Hybridized cellular vesicles are formed by combining materials from various cell types, bacteria [73], or liposomes, resulting in enhanced properties and potential applications in cancer treatment. These vesicles inherit functional attributes from their parental materials, and extensive research has explored the fusion of multiple cell membrane vesicles to achieve diverse functionalities. As the field continues to evolve, hybridized cellular vesicles offer promising prospects for advancing cancer treatment approaches [74,75].

**Figure 4:**
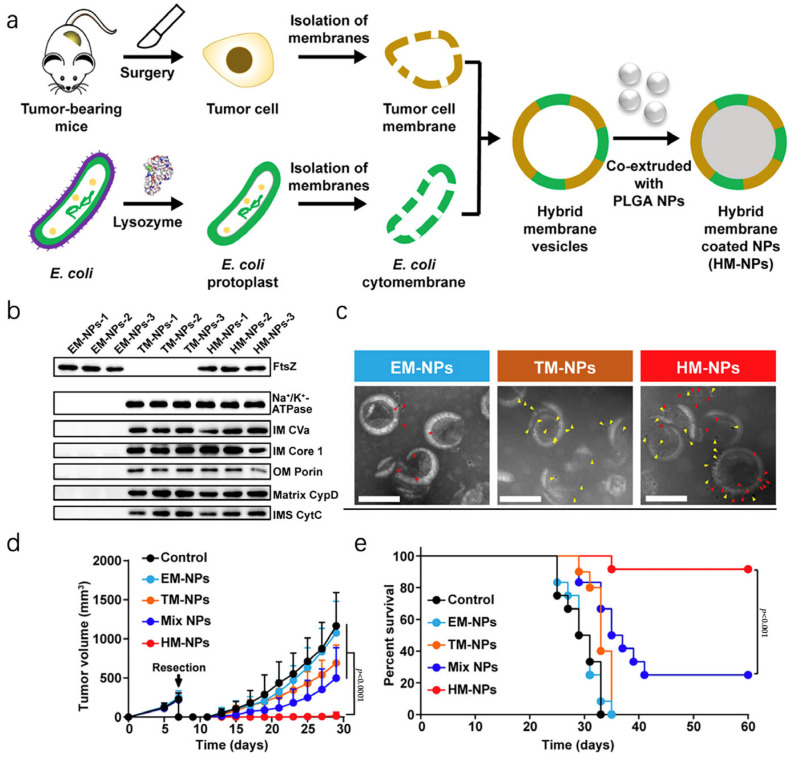
Hybridization of cellular vesicles. (**a**) A diagram illustrating the formation of hybrid vesicles by combining the outer membranes of bacteria and tumor cells. (**b**) In the western blot, membrane proteins were extracted from EM-NPs, TM-NPs, and HM-NPs. (**c**) FtsZ (red arrowheads) and Na^+^/K^+^-ATPase (yellow arrowheads) are immunogold-labeled on EM-NPs, TM-NPs, and HM-NPs in TEM images. Scale bars: 100 nm. (**d**) Curves depicting the progression of the tumor. (**e**) Survival curves depicting the outcomes of the treated and control groups. Reproduced with permission [77]. Copyright 2021, Sci Transl Med.

Bacteria are common pathogens that are eliminated by immune cells to prevent severe infections. In this context, it is also possible to consider them natural substances that enhance the body’s immune system, promote innate and adaptive immune responses, and ultimately combat cancer [76]. This study created a hybrid vaccine by co-extruding nanoparticles with both tumor cell membranes and bacterial cytomembranes (Figure 4a). Figure 4b shows that FtsZ, a well-characterized protein involved in bacterial cell division, was found in both Escherichia coli cytoplasmic membrane nanoparticle vaccines (EM-NPs) and hybrid membrane nanoparticle vaccines (HM-NPs). Additionally, several mammalian membrane proteins were present in tumor cell membrane nanoparticle vaccines (TM-NPs) and HM-NPs, such as Na^+^/K^+^-ATPase, IM CVa, IM Core 1, OM Porin, Matrix CypD, and IMS CytC. Moreover, immunogold staining allowed Escherichia coli cytoplasmic membranes and tumor cell membrane-specific markers to be identified directly on the HM-NPs by measuring Na^+^/K^+^-ATPase and FtsZ levels. This was followed by TEM imaging (Figure 4c). As depicted in Figure 4d,e, in the 4T1 tumor model, it was discovered that fusion vesicles had significantly greater tumor-suppressive effectiveness compared to the mere combination of TM-NPs and EM-NPs. Moreover, the HM-NP vaccine enhanced tumor suppression in mice, leading to a survival rate of approximately 92% within 60 days [77]. For personalized tumor immunotherapy, researchers conducted another study where they combined outer membrane vesicles from bacteria with the membrane of tumor cells, aiming to enhance innate and adaptive immunity simultaneously. An accumulation of fusion vesicles in the inguinal lymph nodes was observed, which exhibited potent inhibitory effects on the growth of tumors and the spread of cancer to lung metastasis [78].

### 3.3. Cellular Vesicles Stimulating Production of Natural Antitumor Substances before Vesicle Extraction

**Figure 5:**
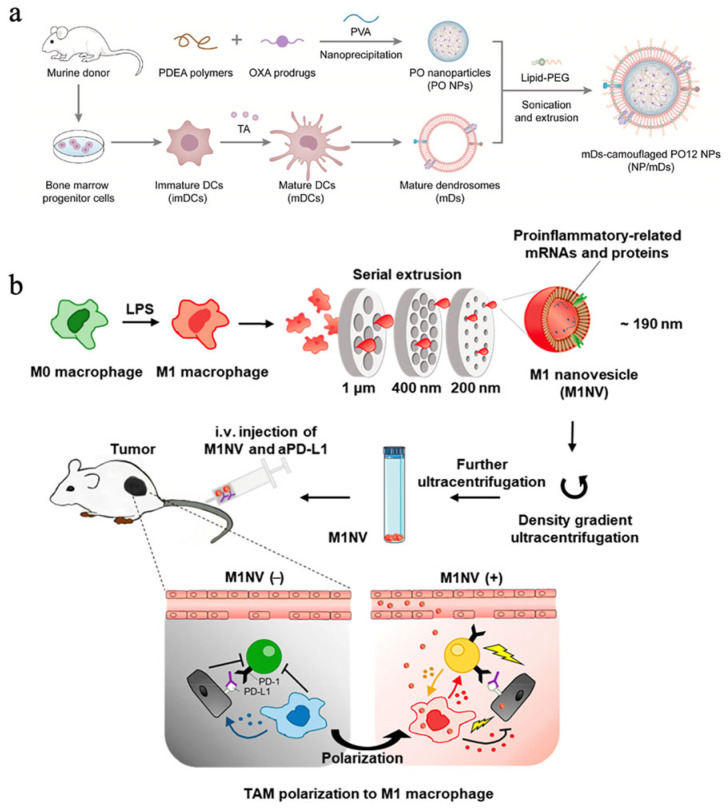
Cellular vesicles stimulate the production of natural antitumor substances prior to vesicle extraction. (**a**) Diagram illustrating the nanoprecipitation of PO NPs and the extraction of mDs, as well as the cloaking of NPs/mDs. (**b**) Diagram illustrating the process of generating nanovesicles from M1 macrophages (M1NVs). On the left, M2 tumor-associated macrophages (TAMs) release anti-inflammatory cytokines that inhibit T-cell activation through aPD-L1 and facilitate the expansion of tumors. M1 macrophages release proinflammatory cytokines when M2 TAMs are polarized by M1NVs, stimulating T cells and initiating an immune response against cancer. Active T cells induce M1 activation by secreting cytokines (on the right). Reproduced with permission [80]. Copyright 2018, ACS Nano, and reproduced with permission [81]. Copyright 2022, Bioactive Materials.

Stimulating immune cells to express endogenous antitumor substances and then extruding vesicles is also an essential strategy. Under suitable preparation, it is believed that activated or polarized immune vesicles retain the mRNA and miRNA contents of the original cells [79]. An intriguing endeavor in the field of research involved the fusion of mature DC vesicles with nanoparticles containing oxaliplatin. Chemotherapy resulted in ICD, and T cells were activated by mature DC vesicles by displaying tumor antigens, thereby enhancing antitumor immunity (Figure 5a) [80]. According to another investigation, the mRNA levels of various pro-inflammatory cytokines were found to be high in nanovesicles derived from M1-type macrophages. As a result of these nanovesicles, macrophages were shown to become polarized towards M1 and CD8^+^ T cells infiltrated into tumors (Figure 5b) [81].

### 3.4. Drug Delivery Nanovectors of Cellular Vesicles

**Figure 6:**
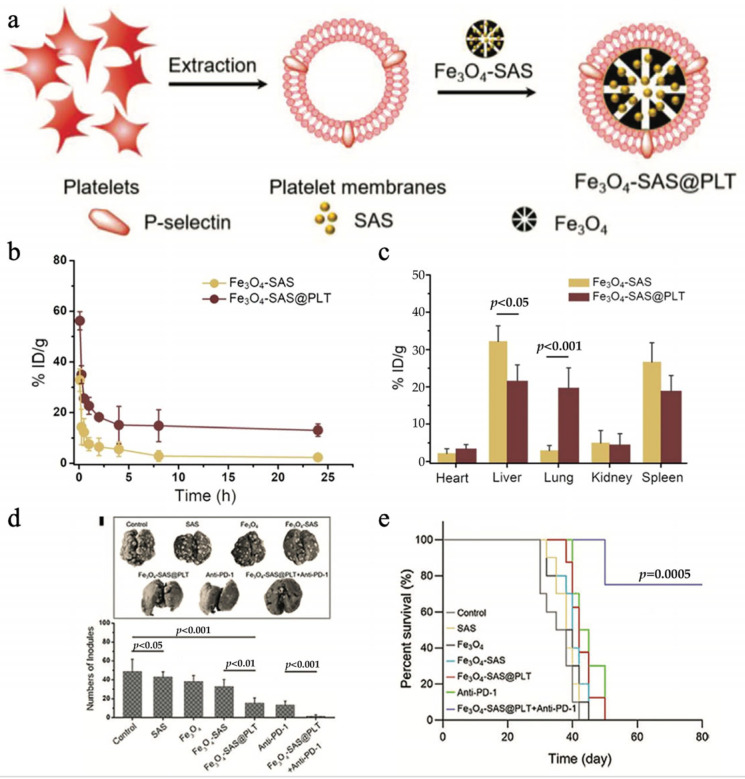
Drug delivery nanovectors of cellular vesicles. (**a**) A diagram illustrating the formation of magnetic nanoparticles camouflaged in platelet membranes. (**b**) Fe_3_O_4_-SAS and Fe_3_O_4_-SAS@PLT in vivo pharmacokinetics. (**c**) An analysis of the biodistribution of Fe_3_O_4_-SAS 24 h after injection and Fe_3_O_4_-SAS@PLT. (**d**) The number of lung metastasis nodules in different groups, as shown in the representative lung photographs. (**e**) Survival curves depicting the outcomes of the different groups. Reproduced with permission [89]. Copyright 2020, Small.

Cellular vesicles, with their hollow-core structure, are widely regarded as an excellent option for delivering drugs because they are non-immunogenic, have low toxicity, and are biocompatible [82]. A combination of cellular vesicles and nanoparticles can be easily mixed and then prepared for transportation into the desired cell membrane-coated nanoparticles using co-extrusion or ultrasonication techniques [83]. Cancer immunotherapy utilizes drugs or biological substances to regulate the immune system and stimulate a suitable immune reaction for the prevention and treatment of cancer. The tumor microenvironment (TME) is a critical factor in tumor immunity and closely influences tumor initiation and progression. It is characterized by vascular abnormalities, low pH levels, and hypoxia, which collectively contribute to immunosuppression. This immunosuppressive TME presents a significant challenge for effective antitumor immunotherapy strategies [84,85]. By remodeling the TME, immune cells can infiltrate more effectively, and tumor growth and metastasis can be suppressed [86,87]. However, precise drug delivery to tumors remains a challenge. Cancer immunotherapy can be effectively pursued through the use of cell-membrane-derived nanoparticles (CMNPs). CMNPs leverage a patient’s tumor cells to create personalized therapy, while their polymer core can hold numerous medications, thereby boosting the immune response against the tumor. Furthermore, CMNPs can envelop immunotherapeutic drugs and employ immune cell membranes derived from leukocytes and cytotoxic lymphocytes to produce immune factors, thereby enhancing their immunotherapy potential [88]. In a study by Jiang and colleagues [89], an innovative strategy was employed to merge mild immunogenic ferroptosis with immune checkpoint blockade therapy targeting PD-1 to treat cancer. Fe_3_O_4_-SAS@PLT nanoparticles were synthesized by loading sulfasalazine (SAS) onto plated membrane (PLTM)-coated Fe_3_O_4_ magnetic nanoparticles. Fe_3_O_4_-SAS@PLT specifically targeted tumors in mice by expressing p-selectin on PLTM, and ferroptosis was induced at the tumor site. As a result, an immune response is activated (Figure 6a). The tumor site continued to show a significant signal even 24 h after injection, indicating that the presence of PLTM enabled Fe_3_O_4_-SAS@PLT to avoid detection by immune cells and prolong its circulation period (Figure 6b,c). Moreover, the immune reaction induced by Fe_3_O_4_-SAS@PLT improved the effectiveness of PD-1 blockers, leading to limited tumor metastasis and extending survival time in mice with metastatic conditions (Figure 6d,e).

## 4. Conclusions and Prospects

There has been a growing interest in vesicles derived from cells in recent years. Cellular vesicles acquire cell membranes and certain cytoplasmic elements and functions from the original cells, rendering them naturally compatible with minimal immunogenicity and toxicity. By altering their genetic makeup, improving their ability to specifically attack cancerous growths and control the immune response in the TME is possible. By combining membranes, innate and adaptive immunity activation can occur simultaneously, offering a promising personalized method for tumor immunotherapy. Compared to cytokine therapy, with which a targeted and high-dose, sustained release of cytokines can be achieved through functional design for rapid tumor treatment, the vaccine strategy based on cell membrane vesicles can target APCs or T cells to deliver tumor-associated antigens, stimulating cytotoxic T cells while also inducing immune memory, thereby more effectively inhibiting tumor recurrence [90]. Moreover, exogenously stimulated cell-derived vesicles containing specific membrane proteins can protect the vesicles from immune clearance. Finally, incorporating drugs into these vesicles establishes a versatile system that merges precise therapeutic administration with immunotherapy, opening up possibilities for cancer theranostics. 

Although cellular vesicles provide notable benefits, incorporating these ideas into practical treatment methods has presented difficulties. The low immunogenicity and high specific targeting of cellular vesicles themselves are crucial when selecting them as biomimetic carriers for cancer immunotherapy, especially in cases where unexpected immune responses may occur. Nevertheless, this presents a necessity for the donor cells to possess a high level of compatibility with the recipient to prevent adverse reactions from the host’s immune system. Furthermore, homotypic recognition by vesicles enables them to migrate toward tumor lesions [91]. At last, it is crucial to determine if external protein alterations impact the equilibrium of the original cells, given that every protein has a distinct function. Additional concerns, including production, retention, durability, and effectiveness, require attention.

Cellular vesicles exhibit immense promise for cancer immunotherapy, and numerous obstacles must be addressed before their extensive use in clinical settings. Key areas of focus for advancing this promising therapeutic approach include guaranteeing the compatibility of donor cells, comprehending the effects of exogenous protein modifications, and addressing concerns related to manufacturing and storage.

## Data Availability

Data are contained within the article.
